# The genome of the polyextremophilic yeast, *Naganishia friedmannii*, reveals adaptations involved in stress response pathways, carbohydrate metabolism expansion, and a limited DNA repair repertoire

**DOI:** 10.1093/femsyr/foaf028

**Published:** 2025-06-05

**Authors:** Lara Vimercati, Clifton P Bueno de Mesquita, Igor V Grigoriev, Sajeet Haridas, Steven K Schmidt, Alisha Quandt

**Affiliations:** Department of Ecology and Evolutionary Biology, University of Colorado, Boulder, CO 80309, USA; The Natural History Museum, Cromwell Rd, South Kensington, SW7 5BD, London 94720, UK; Cooperative Institute for Research in Environmental Sciences (CIRES), Boulder, CO 80303, USA; U.S. Department of Energy Joint Genome Institute, Lawrence Berkeley National Laboratory, Berkeley, CA 94720, USA; U.S. Department of Energy Joint Genome Institute, Lawrence Berkeley National Laboratory, Berkeley, CA 94720, USA; Department of Plant and Microbial Biology, University of California, Berkeley, CA 94720, USA; U.S. Department of Energy Joint Genome Institute, Lawrence Berkeley National Laboratory, Berkeley, CA 94720, USA; Department of Ecology and Evolutionary Biology, University of Colorado, Boulder, CO 80309, USA; Department of Ecology and Evolutionary Biology, University of Colorado, Boulder, CO 80309, USA

**Keywords:** yeast, *Naganishia*, polyextremophile, comparative genomics, high elevation, Volcán Llullaillaco

## Abstract

Here we report the draft genome sequence of *Naganishia friedmannii* (formerly *Cryptococcus friedmannii*) isolate, a Basidiomycota yeast commonly found in some of the most extreme environments of the Earth's cryosphere. We isolated *N. friedmannii* strain Llullensis from soils at 6000 m above sea level on Volcán Llullaillaco, Argentina. The genome was 22.2 Mb with 6251 identified protein coding genes. Proteins known to be associated with thermal, osmotic, and radiation stress were identified in the genome. Comparative analysis with seven other *Naganishia* genomes revealed unique features underlying its polyextremophilic lifestyle. *Naganishia friedmannii* showed an expansion of genes involved in breaking down plant-derived carbohydrates, supporting the hypothesis that it survives at high elevations by metabolizing wind-deposited organic matter. Surprisingly, many genes involved in cell-cycle checkpoints and DNA repair were missing, as in several other *Naganishia* species. This extensive loss may be adaptive in extreme environments prone to abiotic stress, where a high mutation rate could generate advantageous traits, and reduced cell-cycle control may allow for faster reproduction that would be advantageous for rapid growth during brief periods of soil wetting following rare snow events.

## Teaser

The draft genome of *Naganishia friedmannii* strain Llullensis, a basidiomycete yeast isolated from 6000 m high soils on Volcán Llullaillaco, Argentina, reveals adaptations to extreme environments, including stress-related proteins, carbohydrate metabolism genes for survival on wind-blown organic matter, and a surprising loss of DNA repair and cell-cycle checkpoint genes, possibly favoring rapid adaptation and growth in harsh conditions.

## Introduction

*Naganishia friedmannii* is an extremely stress-tolerant Basidiomycota yeast that has been proposed as a model organism for exobiology and studies of stress resistance in eukaryotes (Pulschen et al. 2015, Vimercati et al. 2016). This yeast was first described from endolithic communities in the Dry Valleys of Antarctica (Friedmann 1982, Vishniac 1985) and is abundant in many cryospheric environments including, being the most abundant eukaryotic organism in acidic volcanic soils of the Atacama region (Lynch et al. 2012). Its survival limits have been investigated in a number of studies demonstrating its high tolerance of UV-B and UV-C radiation, extreme temperature fluctuations, desiccation, and exposure to the stratosphere (Pulschen et al. 2015, Vimercati et al. 2016, Pulschen et al. 2018). Recent research has shown that *N. friedmannii* increases in relative abundance in high-elevation volcanic soils when resource limitations are alleviated (Vimercati et al. 2020), suggesting an active role of this microorganism in the extreme environments where it is found. It has been suggested that *N. friedmannii* is an opportunitroph, able to take advantage of brief periods of water availability and metabolize a wide variety of organic compounds (especially carbohydrates) that become available through wind-blown deposition of plant material to high-elevation environments (Schmidt et al. 2017). Closely related members within the *Naganishia* clade have also been isolated from extreme environments, including surfaces aboard the International Space Station (ISS) (Bijlani et al. 2022) and samples from 7900 m.a.s.l. in the Himalayas (Dragone et al. 2023). The analysis of *N. friedmannii*’s genome is important for understanding fungal genome evolution and stress adaptation. Organisms with adaptations to a complex interplay of extreme environmental parameters offer valuable resources for biotechnological exploitation and understanding microbes that are likely to survive future space travel.

We sequenced, assembled, and annotated the genome of *N. friedmannii* strain Llullensis, described and discussed the characteristics of the genome and of the predicted proteome in light of its polyextremophilic nature, and compared the genome to other available genomes in the genus and family. Many basidiomycetous yeasts are adapted to live in environments exposed to multiple stressors and have been detected in a broad range of extreme ecosystems of the cryosphere. However, their adaptations to specific stressors are not well understood. This genome was analyzed with the objective of (i) identifying orthologs of proteins that have been reported to be involved in resistance to environmental stresses; (ii) identifying components of the genome that were expanded or reduced compared to closely related yeasts; (iii) identifying unique components of the *N. friedmannii* genome that are absent from closely related members as well as which components are absent from *N. friedmannii* but present in the others.

## Methods

### Growth conditions and DNA preparation

*Naganishia friedmannii* was isolated from inorganic volcanic soils collected at 6030 m.a.s.l. on Volcán Llullaillaco as described elsewhere (Lynch et al. 2012, Vimercati et al. 2016). Genomic DNA was extracted from mid-exponential cells grown in a liquid medium at the optimal temperature of 17°C containing: 1 g of KH_2_PO_4_; 1 g of MgSO_4_^.^7H_2_O; 0.04 g of NH_4_NO_3_; 0.1 g of carboxymethyl-cellulose; 0.05 g of chitin; 0.1 g of tryptone; 0.1 g of yeast extract per liter of water (Vimercati et al. 2016). The genomic DNA was extracted using a PowerSoil® DNA isolation kit (MOBIO, Carlsbad, CA). We assessed DNA quality and quantity by using the Qubit version 2.0 fluorometer with the Qubit dsDNA HS assay kit (Thermo Fisher Scientific).

### Genome sequencing and assembly

The genome was sequenced on an Illumina MiSeq instrument (250-bp paired-end reads) at the BioFrontiers Sequencing Facility in Boulder, CO. Sequence data was assembled using SPAdes version 3.9.1 (Bankevich et al. 2012) with default settings including adapter removal, trimming, quality filtering and error correction. The genome assembly is publicly available via the Joint Genome Institute's fungal portal MycoCosm (mycocosm.jgi.doe.gov/Nagfr2) (Grigoriev et al. 2014). This Whole Genome Shotgun project has been deposited at DDBJ/ENA/GenBank under the accession JBLXXE000000000. The version described in this paper is version JBLXXE010000000. Additional metadata are available under BioProject accession number PRJNA1179859 and BioSample accession number SAMN44503000 at National Center for Biotechnology Information (NCBI). Summary on genome metrics is provided in Table 1.

**Table 1.** Summary of *N. friedmannii* genome metrics.

Genome assembly size (Mbp)	22.26
Sequencing read coverage depth	229.62x
# of contigs	127
# of scaffolds	103
# of scaffolds ≥2 Kbp	90
Scaffold N50	11
Scaffold L50 (Mbp)	0.65
# of gaps	24
% of scaffold length in gaps	0.1
Three largest scaffolds (Mbp)	2.04, 1.38, 1.29

### Gene prediction and annotation

Structural and functional annotation of the assembly was conducted using the Funannotate pipeline v1.5.2 (Palmer and Stajich 2020), which includes masking (Repeatmasker tool), *ab initio* gene-prediction training using Augustus (with *Cryptococcus neoformans* used as the training species) and Genmark, gene prediction, and the assignment of functional annotation to protein-coding gene models. We compiled a list of proteins from the closest known relatives for the—protein_evidence option. Secondary metabolite (SM) clusters were identified using antiSMASH v5.0 (Blin et al. 2019). Finally, Funannotate “annotate” was run with default settings plus the output from other annotation software programs, including Pfam (Finn et al. 2014), InterProScan 5 (Jones et al. 2014), KEGG (Kyoto Encyclopedia of Genes and Genomes) (Kanehisa and Goto 2000), KOG (Tatusov et al. 2003), and CAZymes (Lombard et al. 2014) to assign functional annotations. Predicted sequences were further annotated with eggNOG v4.0 (Powell et al. 2014).

For a comparative analysis, predictions and annotations were also performed for most closely related genomes available in NCBI within the *Naganishia* species, namely *N. randhawae* eABCC1 (PRJNA634687) (Tshisekedi 2021), *N. albida* JCM2334 (PRJDB3657) (Leo et al. 2023), *N. adeliensis* IF1SW-F1 (PRJNA623412) (Bijlani et al. 2020), *N. liquefaciens* N6 (PRJDB10172) (Han et al. 2020), *N. vishniacii* ANT03-052 (PRJNA196045) (Nizovoy et al. 2021), *N. antarctica* DBVPG 5271 (PRJNA859108) (Turchetti et al. 2022), and *N. tulchinsky* IF6SW-B1(PRJNA623412) (Bijlani et al. 2022). *Naganishia* species have been isolated from diverse terrestrial environments. Table 2 provides an overview of the seven species included in our comparative analysis, outlining their isolation sources, extremophilic habitats, and key morphological adaptations. In addition, genomic sequences of three other related species in the Tremellomycetes class, *Filobasidium wieringae* UCDFST 05–544 (PRJDB3683) (https://mycocosm.jgi.doe.gov/Crywi1), *Solicoccozyma terricola* JCM24523 (PRJNA327103) (Close et al. 2016) and *Cryptococcus neoformans* JEC21(PRJNA10698) (Loftus et al. 2005) were equally processed and used as an outgroup. Comparative genomics was performed using the “funannotate compare” script. The raw Funannotate file is available on Zenodo at DOI: 10.5281/zenodo.14742698. Genome completeness was assessed using BUSCO (Simão et al. 2015) with multiple lineage-specific datasets (Fungi, Basidiomycota, Tremellomycetes, and Eukaryota), with full results presented in Table S1.

**Table 2.** *Naganishia* species included in the comparative analysis, with details on their isolation sources, extremophilic environments, and notable morphological adaptations.

Species	Strain (BioProject ID)	Isolation source	Morphology	Adaptations/notes	Reference
*N. randhawae*	eABCC1 (PRJNA634687)	Tree trunk; avian guano	Spherical-oval, white-cream mucoid colonies	Psychrotolerant; produces extracellular polysaccharides; utilizes various carbon sources	Tshisekedi (2021)
*N. albida*	JCM2334 (PRJDB3657)	Temperate soil; clinical settings	Cream-white, ellipsoidal, encapsulated	Mesophilic; opportunistic pathogen; assimilates inositol	Leo et al. (2023)
*N. adeliensis*	IF1SW-F1 (PRJNA623412)	Antarctic freshwater; sea ice	Cream-pink mucoid colonies; ovoid-ellipsoidal	Psychrophilic; cryoprotectant production	Bijlani et al. (2020)
*N. liquefaciens*	N6 (PRJDB10172)	Alpine glacial soil/meltwater	Butyrous to slightly mucoid colonies; oval cells	Psychrotolerant; gelatin-liquefying; cold-active enzymes	Han et al. (2020)
*N. vishniacii*	ANT03-052 (PRJNA196045)	Antarctic dry valleys mineral soils	Carotenoid pigments; thick-walled, encapsulated	UV/desiccation-tolerant; low-nutrient adaptation	Nizovoy et al. (2021)
*N. antarctica*	DBVPG 5271 (PRJNA859108)	Antarctic soil and rock surfaces	White-cream, encapsulated ovoid cells	Polyols/trehalose production; freeze-thaw/desiccation tolerance	Turchetti et al. (2022)
*N. tulchinskyi*	IF6SW-B1 (PRJNA623412)	Antarctic subglacial brine	Pigmented, ellipsoidal; biofilm-forming	Barotolerant; heavy metal resistance; psychrotolerant	Bijlani et al. (2022)

### Phylogenetic tree reconstruction

In the present study, a set of single copy shared orthologs among the eight *Naganishia* species and the three other Tremellomycetes (*C. neoformans, S. terricola, F. wierigae*) was determined by ProteinOrtho v.6.0.3 (Lechner et al. 2011). Protein sequences were aligned with MUSCLE with default parameters (Edgar 2004) and trimmed with trimAl (Capella-Gutiérrez et al. 2009). Alignments were concatenated and rooted phylogenetic trees were constructed using RaxML-NG v.1.0.1 (Kozlov et al. 2019) with the WAG model of amino acid substitutions. Branch support values were determined using 1000 bootstrap replicates. Genomic sequence divergence between closely related species was estimated using the pairwise distance Kr value within Genome Tools (Gremme et al. 2013).

### Survey of genes involved in extremotolerance

We compiled a list of genes involved in resistance to several stressors associated with the extreme high-elevation environment where *N. friedmannii* is found, including thermal, oxidative, osmotic, and radiation stress. Sequences of the chosen genes were retrieved from the most closely related species available in Uniprot and NCBI and were screened using BLASTp. Identity of putative target proteins was confirmed by reciprocal blastp (e-values < e^−40^). In this survey we used the common human gene names to refer to their yeast homologues, as these names are widely recognized across taxa. Where appropriate, yeast-specific gene names are provided in parentheses to ensure clarity.

### Orthology analysis

Orthologous clustering of the predicted proteome of *N. friedmannii* combined with the proteomes of seven other *Naganishia* species was performed to identify shared as well as unique gene families. A comparison of orthologous proteins enables inference of potential differences in the allocation of cellular resources in *N. friedmannii*.

Analysis of orthologous gene groups was conducted with ProteinOrtho v.6.0.3 (Lechner et al. 2011) and intersections among the genomes were graphed with the ComplexUpset R package (Krassowski 2021). Furthermore, we extracted both InterProScan and Pfam terms that are shared among all *Naganishia* species, those over- or under-represented in *N. friedmannii* and those without homologues in all other *Naganishia* species. Heatmaps were produced with the pheatmap R package (Kolde 2019). Orthogroup assignments for *Naganishia friedmannii* compared to related yeast species are available on Zenodo at DOI: 10.5281/zenodo.14749607.

### Analysis of amino acids usage

Differential usage of amino acids in *N. friedmannii* compared to its closest relatives was computed using 1745 single copy orthologs common to all species, identified and aligned as described above. The seven other *Naganishia* species as well as *F. wieringae, S. terricola*, and *C. neoformans* were used for the comparison. Alignments were concatenated and gaps eliminated. Comparisons of amino acid composition were made using a Composition Profiler with default parameters (Vacic et al. 2007).

### Positive selection (*d*N/*d*S ratio) analysis

Positive selection of genes can be inferred from a higher proportion of nonsynonymous (*d*N) over synonymous substitutions per site (*d*N/*d*S >1). The *d*N/*d*S ratio was computed for all the single copy orthologs of *N. friedmannii* and *N. randhawae*, as well as all those common between *N. friedmannii* and *F.wieringae*. Gap-free codon alignments of proteins were obtained with PAL2NAL (Suyama et al. 2006) and *.axt files were generated and analyzed within KaKs Calculator Version 2.0. (Wang et al. 2010). *d*N/*d*S ratio analysis was run both for the whole sequences and on 102 nucleotide-length chunks with a sliding window strategy (24 nucleotide steps) using a YN model (Yang et al. 2000, Nizovoy et al. 2021). Signals of positive selection were considered only if the *d*N/*d*S ratio >1 and *p*-value <.05 were shared for the same region on a sequence among *N. friedmanni* and *N. randhawae* and *N. friedmannii* and *F. wieringae*.

### Survey of gene loss in DNA repair and cell-cycle control

We focused on the KEGG pathways for cell cycle and DNA repair due to their critical roles in maintaining genomic stability, as these pathways are generally expected to be well-conserved in organisms exposed to harsh environmental conditions. To determine the presence and absence of genes involved in DNA repair and cell cycle control, we used the list of genes in KEGG pathways of cell cycle for yeast (04111) and pathways within the replication and repair category (03410, 03420, 03430, 03440, 03450). These pathways provide a well-established framework based on the extensively studied *Saccharomyces* species, though we recognize that their annotations may not fully encompass the diversity of cell cycle genes in more distantly related yeasts, including *Naganishia*. Each gene within the pathways of interest was searched in Uniprot and the relative sequence for *S. cerevisiae* and *C. neoformans* was used to search for the corresponding homolog in each *Naganishia* genome through both annotated protein sets and raw genomic scaffolds, ensuring absences were not due to annotation gaps. We also looked for the presence of meiosis-specific genes hypothesized to only be present in organisms with sexual ancestry (Malik et al. 2008). NCBI's blastp function and an e-value cutoff of 1 × 10^−3^ was used, as recommended for homology searches (Pearson 2013).

## Results

### General features of *N. friedmannii* genome

**Figure 1.**
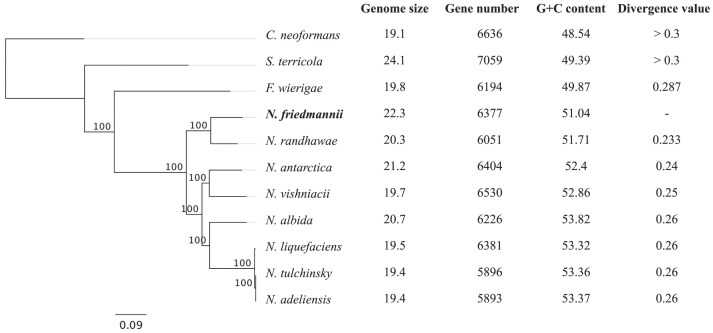
Phylogenetic tree of closest sequenced relatives to *N. friedmannii*, rooted with *Cryptococcus neoformans*. The tree was constructed from 1745 single copy ortholog protein sequences using RAxML-NG v. 1.0.1 with the WAG model of amino acid substitutions. All nodes showed 100% bootstrap support. Divergence values indicate pairwise sequence similarity percentages.

The genome assembly of *N. friedmannii* yielded a 22.26 Mb genome with a 229.6-fold depth of coverage. The assembly resulted in 127 contigs and 51.04% GC content (Table 1). The *N. friedmannii* genome is similar in size to other members of the *Naganishia* genus. All *Naganishia* genomes analyzed have a higher GC content (51.04%–53.82%) than that of closest relatives (Fig. 1).

**Figure 2.**
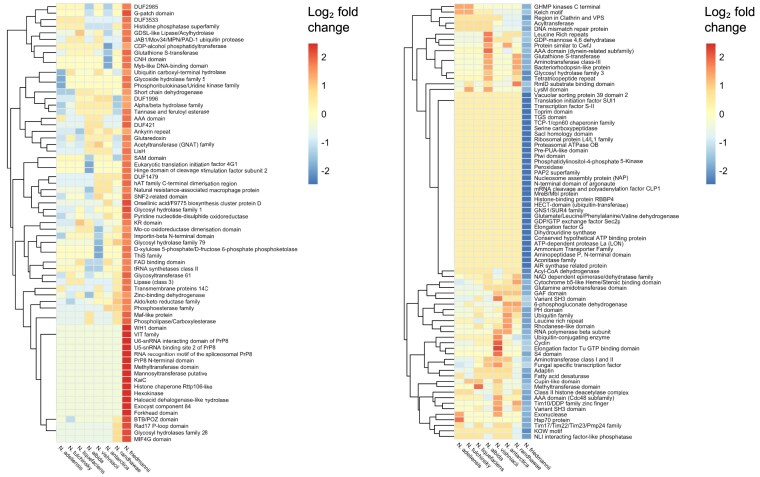
(a) Heatmap of Pfam families whose copy number was the highest in *N. friedmannii* compared to all other *Naganishia* species analyzed. (b) Heatmap of Pfam families whose copy number was the lowest in *N. friedmannii* compared to all other *Naganishia* species analyzed. Dendrogram on the left was generated using hierarchical clustering.

The funannotate pipeline predicted 6377 coding genes. For all the predicted proteins, 3221 were mapped in the KEGG database, and 5164 were classified in the Clusters of Orthologous Groups for Eukaryotes (KOG) database. A total of 1168 KOG predicted genes are of unknown function (22.6%). 4757 predicted proteins contained at least one InterProScan annotated domain and 3023 different Pfam domain assignments were obtained (Tables S2 and S3). A total of 77 Pfam domains were expanded in *N. friedmannii* and 94 were reduced (excluding absent Pfams) (Fig. 2a and b) compared to other species in the genus. 191 InterProScan and 106 Pfam domains were present in other *Naganishia* species but had no counterpart in *N. friedmannii* (Tables S4 and S5).

Carbohydrate-active enzymes (CAZymes) are involved in the biosynthesis and metabolism of carbohydrates and glycoconjugates (Cantarel et al. 2009). A total of 173 CAZymes were identified in the predicted protein set of *N. friedmannii* (Table S6), with GH and GT being the most abundant families with 81 and 51 CAZymes, respectively.

We identified only five SM clusters, comparable to the number found in the other *Naganishia* species (5–7): two Terpenes, two NPRS-like clusters, and one Type 3 PKS cluster (Table S7). 126 tRNA genes were identified, the lowest number within the *Naganishia* genomes analyzed to date.

### Survey in genes involved in extremotolerance

Our analysis of *N. friedmannii* genome identified multiple genes associated with stress response pathways (Table 3), including those linked to temperature, osmotic, oxidative, and general stress. While these genes are not exclusive to *N. friedmannii*, this section focuses solely on their presence and roles in this strain. Among the characteristic temperature stress response proteins, we identified a number of heat shock proteins and chaperones, such as Hsp70 (Ssa1), Hsp90 (Hsp82), GroES (Hsp10), and Cpn60 (Hsp60), which are important for proper folding and function of proteins. *N. friedmannii* also has a gene with homology to an ice-binding protein (IBP). In the osmotic stress category, we identified genes related to the production and regulation of compatible osmolytes, such as trehalose. Trehalose phosphatase had two copies in both *N. friedmannii* and *N. randhawae* compared to the rest of *Naganishia* species with only one gene copy. Several genes involved in biosynthetic pathways of protective compounds against oxidative damage were identified, such as the complete set of genes involved in the synthesis of carotenoids, as well as several involved in the synthesis of mycosporin, astaxanthin, and melanin.

**Table 3.** Identification of stress-related genes in *N. friedmannii* and number of InterProScan predicted gene copies.

Iprscan	Copy number	Gene/domain	Stress type	Pathway/functional role
IPR005804	3	Fatty acid desaturase domain	Temperature	Fatty acid desaturation
IPR001404	2	Heat shock protein Hsp90 family	Temperature	Molecular chaperone
IPR013126	7	Heat shock protein 70 family	Temperature	Molecular chaperone
IPR020818	1	GroES chaperonin family	Temperature	Molecular chaperone
IPR002423	8	Chaperonin Cpn60/TCP-1 family	Temperature	Molecular chaperone
IPR000740	2	GrpE nucleotide exchange factor	Temperature	Protein folding/Heat shock response
IPR021884	1	Ice-binding protein like	Temperature	Freezing inhibition
IPR025279	1	Stress response protein NST1	Temperature	Heat stress response
IPR003337	2	Trehalose-phosphatase	Osmotic	Trehalose metabolism
IPR001830	2	Trehalose-phosphate synthase	Osmotic	Trehalose metabolism
IPR011120	1	Neutral trehalase Ca2 + binding	Osmotic	Trehalose metabolism
IPR003718	1	OsmC/Ohr family	Osmotic	Osmotic stress response
IPR038783	1	Fungal MAP kinase Hog1	Oxidative	Osmotic stress response
IPR005746	2	Thioredoxin	Oxidative	Antioxidant role
IPR000889	2	Glutathione peroxidase	Oxidative	Antioxidant role/Detoxification
IPR002060	2	Squalene/phytoene synthase	Oxidative	Carotenoid synthesis
IPR004294	2	Carotenoid oxygenase	Oxidative	Carotenoid metabolism
IPR030960	2	2-epi-5-epi-valiolone synthase	Oxidative	Mycosporine synthesis
IPR002935	2	Catechol o-methyltransferase	Oxidative	Mycosporine synthesis
IPR011761	10	Carbamoylphosphate synthase large subunit	Oxidative	Mycosporine synthesis
IPR011876	1	Isopentenyl-diphosphate delta-isomerase	Oxidative	Astaxanthin synthesis
IPR008930	4	Farnesyltranstransferase	Oxidative	Astaxanthin synthesis
IPR000092	3	Farnesyl pyrophosphate synthetase	Oxidative	Astaxanthin synthesis
IPR036396	8	Astaxanthin synthase	Oxidative	Astaxanthin synthesis
IPR033138	3	Laccase	Oxidative	Melanin synthesis
IPR012328	1	Polyketide synthase type III	Oxidative	Melanin synthesis
IPR002109	7	Glutaredoxin	Oxidative	Antioxidant role/Detoxification
IPR040079	5	Glutathione Transferase family	Oxidative	Antioxidant role/Detoxification
IPR011941	1	DNA recombination/repair protein Rad51	Oxidative	DNA repair
IPR041247	1	Rad52 family	Oxidative	DNA repair
IPR023167	1	Yap1 redox domain superfamily	Oxidative	Oxidative stress response
IPR001424	1	Superoxide dismutase, copper/zinc binding domain	General	Antioxidant role
IPR011614	3	Catalase core domain	General	Antioxidant role
IPR009053	5	Prefoldin	General	Protein folding
IPR012724	2	Chaperone DnaJ	General	Molecular chaperone
IPR002828	2	Survival protein SurE	General	Environmental stress response
IPR003021	1	Rad1	General	Cell cycle checkpoint
IPR007268	1	Rad9/Ddc1	General	Cell cycle checkpoint
IPR012725	1	DNA K	General	Molecular chaperone

### Expansions and contractions of protein families

**Figure 3.**
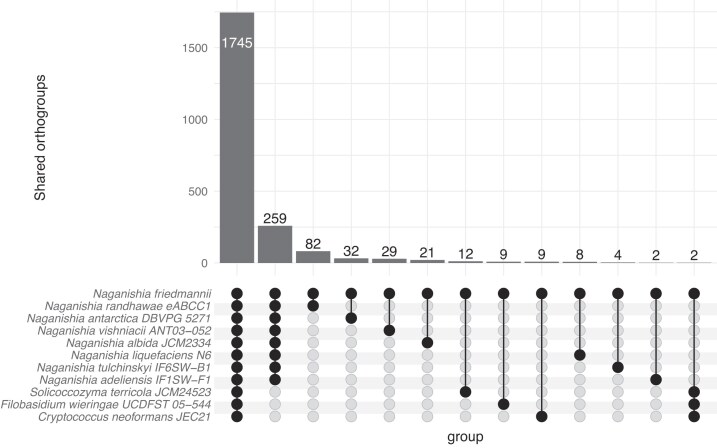
Number of shared orthologs among eight *Naganishia* species and selected closest relatives within the class Tremellomycetes. Bars represent the number of genes within shared orthogroups. Species overlaps are shown among all *Naganishia* species and for *N. friedmannii* with other individual *Naganishia* species.

**Figure 4.**
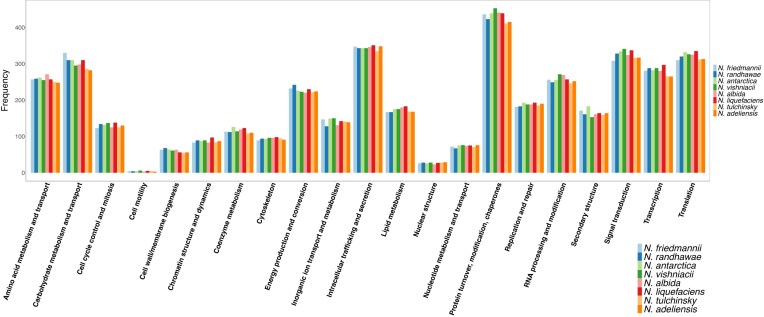
Comparison of the frequency of KOG functional classifications among eight *Naganishia* species.

The identification of orthologs shared between *N. friedmannii* and seven other available *Naganishia* genomes and three other Basidiomycota yeasts enabled us to contrast the contents of these genomes (Figs 1 and 3), thus identifying expanded and reduced protein families in *N. friedmannii*, as well as absent and unique proteins. Orthology inference analysis revealed that 1745 was shared between *N. friedmannii* and all other Tremellomycete species considered in this study (Fig. 3). Only 259 orthogroups were common to just the eight *Naganishia* species. Among the protein families that exhibited expansion, the most abundant were related to the carbohydrate metabolism and transport KOG category, such as glycosyltrasferases and glycosil hydrolases (Figs 2a and 4). Among protein families in *N. friedmannii* that exhibited the most differences in copy number compared to the other *Naganishia* species, we found tannases/feruloyl esterases and xylulose 5-phosphate phosphoketolase (Fig. 2a). Major facilitator superfamily (MFS) and MFS transporter had the highest copy number according to InterProScan (Table S2). Reduced families belonged to several different GO categories including some families associated with stress responses in fungi such as Hsp70, Cpn60, DNA mismatch repair protein, glutathione-S-transferase, and peroxidase (Fig. 2b).

Out of the predicted 6251 putative protein coding sequences for *N. friedmannii*, ∼12% of the genes (746) were unique to the *N. friedmannii* genome. The majority of these annotated singleton orthologs fell into the carbohydrate metabolism and transport category (Fig. S1). A total of 106 Pfam terms that were annotated in all *Naganishia* species analyzed were absent from *N. friedmannii* (Table S5). Among these, we discovered the absence of a number of genes associated with DNA replication, repair and cell cycle control such as DNA topoisomerase IA, Rad4, Spo12, APC10, and Mlh1 C-terminus. In addition, we found the absence of a number of Pfam terms associated with proteins involved in intracellular transport, such as coatomer and clathrin sunbunits, Sec8 and VPS28. A number of Pfam terms associated with ribosomal proteins were also missing.

### Analysis of amino acids usage

**Figure 5.**
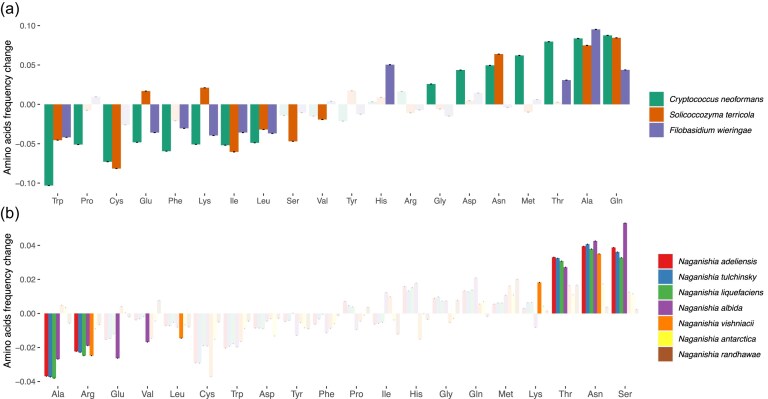
Amino acid frequency changes in *N. friedmannii* with respect to *C. neoformans, S. terricola* and *F. wieringae* (a) and with respect to other *Naganishia* species (b) tested in single copy shared proteins. Highlighted bars show amino acids with significant differences. Standard deviation is shown for each bar. Exact values are provided in Table S7.

We computed differences in amino acid usage using single-copy orthologous genes of the *Naganishia* species as well as closely related members within the Tremellomycetes. Initial comparison of *N. friedmannii* versus outgroup species (*C. neoformans, S. terricola, F. wieringae*) revealed a significant enrichment of alanine and glutamine alongside a significant depletion of bulky residues such as tryptophan, isoleucine and leucine (Fig. 5a and Table S8). Expanding this analysis to the entire *Naganishia* clade confirmed consistent enrichment of small/flexible residues (alanine, glutamine) across species but showed broader variation in bulky amino acid depletion patterns (Table S9). No significant differences in amino acid composition were observed between *N. friedmannii* and *N. randhawae* and *N. antarctica*. Enrichment in asparagine and depletion in arginine was observed when *N. friedmannii* was compared with the other five *Naganishia* species (Fig. 5b, Table S8).

### *d*N/*d*S ratio analysis

**Figure 6.**
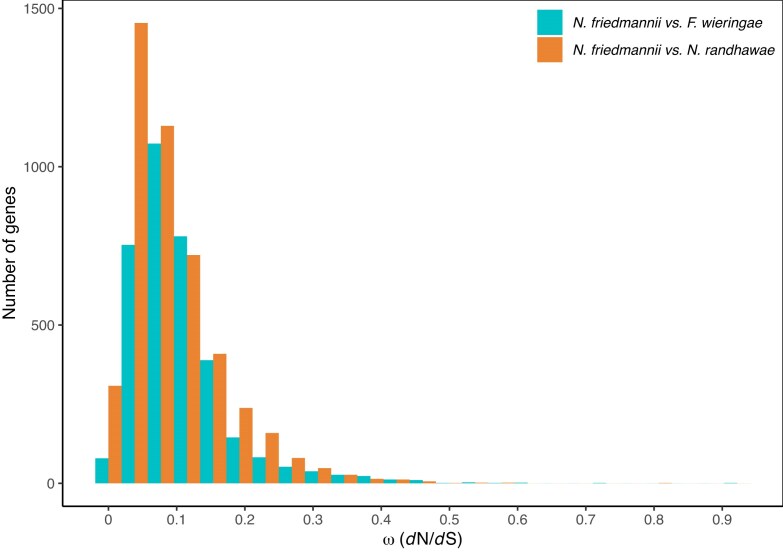
Distribution of *d*N/dS ratios for pairwise orthologous protein coding sequences between *N. friedmannii* and *F. wieringae* (3472) and *N. friedmannii* and *N. randhawae* (4609). No genes show a *d*N/*d*S ratio ≥1 suggesting they are under purifying or relaxed selection regimes.

The distribution of *d*N/*d*S mean values for the 3472 single copy orthologs between *N. friedmannii* and *F. wieringae* ranged from ∼0 to 0.92 and 92% of genes had a *d*N/*d*S <0.2, while the distribution of *d*N/*d*S mean values for the 4609 single copy orthologs between *N. friedmannii* and *N. randhawae* ranged from ∼0 to 0.8 and 91% of genes had a *d*N/*d*S <0.2 (Fig. 6). None of the proteins analyzed had a *d*N/*d*S mean ratio >1. However, the calculation of *d*N/*d*S by means of a sliding window strategy detected six proteins whose value was ∼1 in shared regions between *N. friedmannii* vs *N. randhawae* or *F. wieringae*. These proteins were annotated as: isocitrate dehydrogenase, ribosome biogenesis protein tsr3, PI31 proteasome regulator, chitin deacetylase, and hypothetical protein NCC49.

### DNA repair and cell-cycle control gene loss

**Figure 7.**
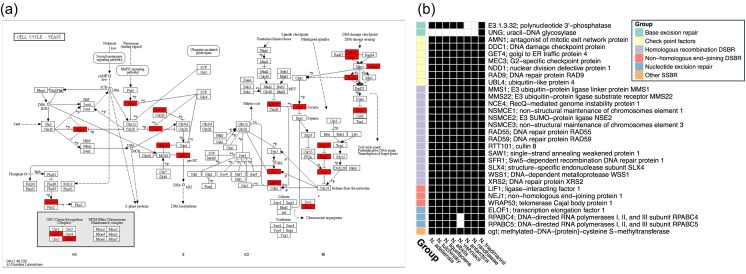
Presence/absence pattern of (a) cell cycle genes and (b) DNA repair genes in *Naganishia* species. (a) Genes shown in red are from the KEGG pathway ‘ko04111 Cell Cycle—Yeast’ and are absent in the *N. friedmannii* genome. Pathway data were sourced from the KEGG database (Kanehisa et al. 2021). (b) Genes associated with DNA repair are shown. Genes represented are absent at least in one other *Naganishia sp*. Black squares indicate absence of the gene in the species shown. Selected genes were obtained from the KEGG DNA Repair and Recombination Proteins—*Saccharomyces cerevisiae* (budding yeast). DSBR and SSBR refer to double-strand and single-strand break repair mechanisms, respectively.

Across the entire *Naganishia* genus, we observed consistent losses in DNA repair and cell-cycle control genes. Specifically, *N. friedmannii* is lacking 20/125 cell cycle genes and 23/154 DNA repair genes as identified with KEGG using *C. neoformans* and *S. cerevisiae* as reference (Fig. 7a and b and Tables S10 and S11). These losses are not strain-specific: all other *Naganishia* species share the absence of every cell cycle control gene missing in *N. friedmannii*, except for anaphase-promoting complex subunit 10 (APC10) and sporulation-specific protein SPO12. Similarly, DNA repair gene losses are genus-wide, with only seven exceptions: MGMT (MGT1), UNG, RPABC4 (RPC19), RPABC5 (RPC5), RAD59, NSMCE2 (MMS21), and NSMCE3 (NSE3). *Naganishia friedmanni* possesses most of the conserved meiosis-specific gene homologues analyzed, except for Hop2, Rec8, and Pds5 (Table S12).

## Discussion

### General features of *N. friedmannii* genome

The genome of *N. friedmannii* was sequenced and analyzed to understand its polyextremophilic lifestyle. The analysis revealed that genome metrics are in line with other members of the genus *Naganishia*. Compared to other *Naganishia* species, *N. friedmannii* possesses the largest number of genes related to carbohydrate metabolism and transport (Fig. 4) and has the lowest GC content. While high GC content is typically a feature of psychrophilic fungi, aiding in DNA stabilization following damage (Gomez et al. 2024), the lower GC content in *N. friedmannii* probably reflects its adaptation to habitats with a wide temperature range (Lynch et al. 2012). *N. friedmanni* is most closely related to *N. randhawae*, which was isolated for the first time from avian guano and is a putative pathogen (Tshisekedi 2021). *N. friedmannii* shares several genes associated with virulence factors found in the closely related pathogen *C. neoformans* (Fu et al. 2022, Al-Huthaifi et al. 2024). These include genes for capsule biosynthesis (CAP59 and CDA), melanin biosynthesis genes (TYR1 and PKS) and thermotolerance genes (HSP90). However, virulence is influenced by a complex interplay of multiple factors, making it challenging to assess the potential pathogenicity of a strain based solely on the presence or absence of specific genes. Further research will be necessary to better understand the pathogenic potential of *N. friedmannii*.

### Survey of genes involved in extremotolerance

The investigation for genes related to stress response and tolerance in *N. friedmannii* revealed the presence of a wide range of mechanisms that help explain its polyextremophilic lifestyle (Table 3). A wide temperature range characterizes the environment from where *N. friedmanni* strain Llullensis was isolated from, which can go from a low of −7°C to a high of 42°C (measured at 4 cm depth in the soil) within 24 h. In laboratory experiments, *N. friedmannii* survived daily fluctuations from −10°C to 30°C and actively grew under those conditions (Vimercati et al. 2016). Mechanisms to counteract temperature extremes include the synthesis of heat shock proteins, membrane fluidity regulations, and molecular adaptations to enable functioning in extreme heat or cold (Alcaíno et al. 2015, Abu Bakar et al. 2020). The genome of *N. friedmannii* is endowed with several heat-shock proteins and molecular chaperones as well as a general stress protein and fatty acid desaturase genes (Table 3). An important adaptation is the protection against the risk of freezing during extreme thermal fluctuations, which can be achieved by producing antifreeze (AFP) and ice-binding (IBP) proteins. Homology search for IBP in all *Naganishia* spp. yielded positive results in *N. friedmannii* and *N. antarctica* genomes only. *Naganishia friedmannii* has a gene with a high homology (2e^−61^) to an antifreeze protein in the distantly related Basidiomycota yeast, *Glaciozyma antarctica* (Hashim et al. 2013, Firdaus-Raih et al. 2018). Interestingly, it has been proposed that ice-binding proteins in psychrophilic fungi and algae could have originated from horizontal gene transfer from bacteria (Arai et al. 2019, Dorrell et al. 2023).

*Naganishia friedmannii* harbors genes crucial in trehalose biosynthesis and degradation. Trehalose is a disaccharide that helps protect the integrity of cells against a variety of environmental stresses such as desiccation, dehydration, heat, cold, salt, and oxidation (Argüelles et al. 2017). Cells accumulate trehalose upon severe cold shock, and it has been shown that it is critical for the cell's resistance to freezing and maintenance of viability at 0°C or lower (Inouye and Phadtare 2004). Upon return to normal temperatures, trehalose levels rapidly decrease as a result of degradation by the neutral trehalase enzyme (Kandror et al. 2004). Previous work with *N. friedmannii* strain Llullensis showed that it can also grow using trehalose, a carbon and energy source (Vimercati et al. 2016), further emphasizing the metabolic versatility of this organism.

Regarding oxidative stress, *N. friedmannii* possesses genes involved in several biosynthetic pathways for protective molecules such as astaxanthin, mycosporine, melanin, and carotenoids. These photoprotective compounds are produced by several yeasts isolated from similar extreme environments (Vaz et al. 2011, Rosa 2019). Genes associated with their biosynthesis have recently been identified in the closely related *N. vishniacii* (Nizovoy et al. 2021), although experimental evidence of their production in this species is still lacking. These compounds serve as UVB-screening agents with antioxidant properties (Moliné et al. 2010), which are essential in environments with high solar irradiation, such as the high elevations of the Atacama Desert where *N. friedmannii* is abundant.

### Expansions and contractions of protein families

The most significant protein families that exhibited expansion in *N. friedmannii* are enzymes involved in the KOG functional category of carbohydrate metabolism and transport (Fig. 4). In addition, 110 out of 262 CAZymes identified in the genome are glycoside hydrolases (GHs), indicating a high capacity to break down simple and complex carbohydrates. This finding supports previous phenotypic results that showed that *N. friedmannii* can utilize a wide variety of complex (e.g. arbutin, cellobiose, gentibiose, melezitose, dextrin, salicin, trehalose etc.) and simple (e.g. arabinose, galactose, xylose etc.) carbohydrates as growth substrates (Vimercati et al. 2016). Family expansions of GHs as well as MFS transporters have been reported before for psychrophilic Tremellomycetes such as *D. cryoxerica* and *M. psychrophila* (Su et al. 2016) and Dothideomycetes (Gomez et al. 2024). Under extreme stress, such as conditions found in environments where *N. friedmannii* is found, microorganisms have difficulty in accessing nutrients and these types of transporters might help in maintaining the consistency of nutrient uptake and energy availability.

Tannases were also significantly enriched within the *N. friedmannii* genome. These inducible enzymes are involved in degrading plant matrices such as lignin to produce gallic acid and glucose and modifying phenolic molecules, facilitating their solubility and metabolism (Hur et al. 2014). These findings support the hypothesis that *N. friedmannii* is well adapted to opportunistically take advantage of wind-blown deposited plant material to grow during rare periods of water availability in its high-elevation habitat (Vimercati et al. 2016, Schmidt et al. 2017). Xylulose 5-phosphate phosphoketolase was present in twice as many copies in *N. friedmannii* compared to other *Naganishia* species. Phosphoketolases facilitate a catabolic alternative to the canonical pentose phosphate pathway and are crucial for sugar-phosphate metabolism in obligate and heterofermentative bacteria, as well as in certain species of microalgae, cyanobacteria, and fungi (Sánchez et al. 2010). The thermotolerance and ability to degrade a wide range of carbohydrates and related compounds make *N. friedmannii* a promising candidate for the bioconversion of recalcitrant biomass into valuable products.

Surprisingly, a number of proteins well known for their role in stress responses were contracted in *N. friedmannii* compared to other *Naganishia* spp., such as Hsp70, LON protease, peroxidase, fatty acid desaturase and several proteins involved in DNA repair and cell cycle. This suggests that adaptation to extreme and unstable environments may be favored by a diversified set of stress response strategies, rather than just relying on a few specific sets of proteins to cope with stress (Gomez et al. 2024). Only five SM clusters were found within the *N. friedmannii* genome. Previous research has pointed out that genome size and number of SMs tend to be decreased in oligotrophic fungi, including some other psychrophilic Basidiomycota species (Batista et al. 2020). SM production is thought to provide a competitive edge in environments with high microbial activity, but it may be less beneficial in extreme or oligotrophic environments where fewer species are present. Research indicates that in nutrient-limited conditions, many organisms display neutral or limited interactions with their co-existing microorganisms (Velez et al. 2018, Yan et al. 2021). Additionally, SMs are known to act as signaling molecules, mediating interactions between fungi, their surroundings, host plants, and other microbes (Elhamouly et al. 2022, Yu et al. 2023). In oligotrophic environments, where fewer ecological interactions occur, it would therefore be adaptive for organisms like *N. friedmannii* to reduce SM production, conserving energy and resources.

The *N. friedmannii* genome contains ∼12% unique proteins compared to other *Naganishia* species. This high number of unique proteins can be explained by the fact that fungi are often highly divergent at the genome level, even within members of the same species (Galagan et al. 2005). Interestingly, a number of transposable element genes were present in the *N. friedmannii* genome but absent in all other *Naganishia* spp., such as Kyakuja-Dileera-Zisupton transposase (IPR040521) and Transposon En/Spm-like (IPR004242). It has been reported that the activity of transposable elements enables species to rapidly adapt to challenging environmental conditions by causing rearrangements within the genome (Schrader and Schmitz 2019). This mechanism has been observed in closely related species such as *C. deneoformans* (Gusa et al. 2023), and it is a strategy that *N. friedmannii* might also employ.

### Analysis of amino acids usage

The comparison of amino acid frequency across *Naganishia* clade species and their closest non-*Naganishia* relatives revealed a significant enrichment of glutamine and alanine, suggesting these modifications are a conserved clade-wide adaptive strategy. Amino acids have been described according to their flexibility index (Li et al. 2006, Rao et al. 2011). Glutamine is a flexible amino acid and alanine is the smallest amino acid. Increased usage of glutamine has been reported before in psychrophilic yeasts related to *N. friedmannii* (Nizovoy et al. 2021). High flexibility has been suggested as one of the main structural features of cold-adapted proteins to compensate for lower chemical reaction rates at low temperatures (Gerday et al. 2000, Gianese et al. 2001). Conversely, some larger and less flexible amino acids that restrict backbone rotations (e.g. tryptophan, isoleucine, leucine) are depleted in *N. friedmannii*. This pattern of bulky residue depletions is conserved across the *Naganishia* clade when compared to outgroups (*C. neoformans, S. terricola, F. wieringae*), though with some lineage-specific variation in both the degree and identity of depleted residues suggesting lineage-specific adaptations. Additionally, *N. friedmannii* does not possess the complete biosynthetic pathways required for synthesizing the amino acids lysine, phenylalanin, histidine, and tryptophan. Many fungi with reduced genomes have similarly lost genes related to amino acid biosynthesis as an evolutionary adaptation (Guedes et al. 2011). The production of amino acids, such as lysine and tryptophan, is energetically costly and involves multiple enzymatic steps. By discarding the genes associated with these pathways, *N. friedmannii* can reduce its genetic burden and instead acquire amino acids from wind-blown deposited materials in its environment​.

Overall, an amino acid profile with a significant preference for flexible and small amino acids and depletion of larger ones may be a key feature in *N. friedmannii* to both facilitate catalytic activity at low temperatures and decrease the energetic cost of conformational change required in fast changing temperature environments such as the dramatic 40°C changes in 12 h observed on Volcán Lllullaillaco.

### *d*N/*d*S ratio analysis

Gene-wide *d*N/*d*S analyses revealed no evidence of positive selection. However, a sliding window approach identified localized regions within six genes where *d*N/*d*S values exceeded 1, suggesting potential site-specific positive selection. Two of these genes fall into the KOG functional categories of carbohydrate and amino acid metabolism (chitin deacetylase and isocitrate dehydrogenase). Earlier work has shown that chitosan (the end product of chitin deacetylase) is necessary for cell wall integrity in the related *C. neoformans* (Baker et al. 2007). The higher levels of non-synonymous mutations in these genes may be driven by divergent selection from the harsh high-elevation desert conditions that characterize the habitat of *N. friedmannii*. The ribosome biogenesis protein tsr3 is responsible for the complex modification of the P-site in yeast 18S ribosomal RNA, and our results suggest ribosomal function may be under selection in *N. friedmannii*.

### DNA repair and cell-cycle control gene loss

Genes involved in cell-cycle control and DNA repair processes are highly conserved across the tree of life and fundamental for genome integrity. Loss of these genes was until recently considered extremely rare and only observed in cancer cells (Hartwell 1992). However, recent investigations of different fungal lineages have uncovered how extensive losses or mutations within these gene categories may not be uncommon in the fungal kingdom (Milo et al. 2019, Steenwyk et al. 2019, Phillips et al. 2021), including hypermutant pathogenic strains within the *Cryptococcus* genus (Billmyre et al. 2017, Boyce et al. 2017), a yeast genus closely related to *Naganishia*. Genome reduction is a well-documented strategy among extremophiles, where energy conservation and functional efficiency often outweigh the benefits of genomic redundancy (Rappaport and Oliverio 2023). In *N. friedmannii*, this manifests as a retention and expansion of metabolic functions—such as carbohydrate metabolism—alongside the loss of environmentally dispensable genes, including certain DNA repair pathways. This trade-off is potentially ecologically strategic: *N. friedmannii*’s expanded carbohydrate metabolism likely supports survival in oligotrophic conditions, enabling the breakdown of scarce organic polymers, while repair gene loss may accelerate adaptive evolution through increased mutation rates. Loss of function in one or few of cell-cycle control or DNA repair genes can lead to a dramatic increase in the mutation rates, display higher rates of genomic evolution at the nucleotide level and cause a more rapid adaptation to novel stressors *in vitro* (Billmyre et al. 2020) and to novel environments (Priest et al. 2020).

All the *Naganishia* species analyzed have undergone a substantial loss of DNA repair genes indicating that this may be a pattern across this lineage spanning multiple species. Notably, most *Naganishia* species are found in extreme environments, particularly cold regions such as Antarctica or high-altitude ecosystems, suggesting that impaired DNA repair mechanisms may represent an evolutionary adaptation to harsh conditions characterized by high UV radiation, temperature variability, and other environmental stresses. As a result, it is possible that these strains have the ability to experience elevated genome-wide mutation rates, which can provide fitness benefits in stressful or changing conditions. To determine the evolutionary impact of DNA repair gene loss, it will be necessary to experimentally verify whether *N. friedmannii* and related *Naganishia* spp. exhibit increased mutation rates. Isolating more strains of *N. friedmannii* would also enable population genetic assessments of the variation seen in natural populations.

**Figure 8.**
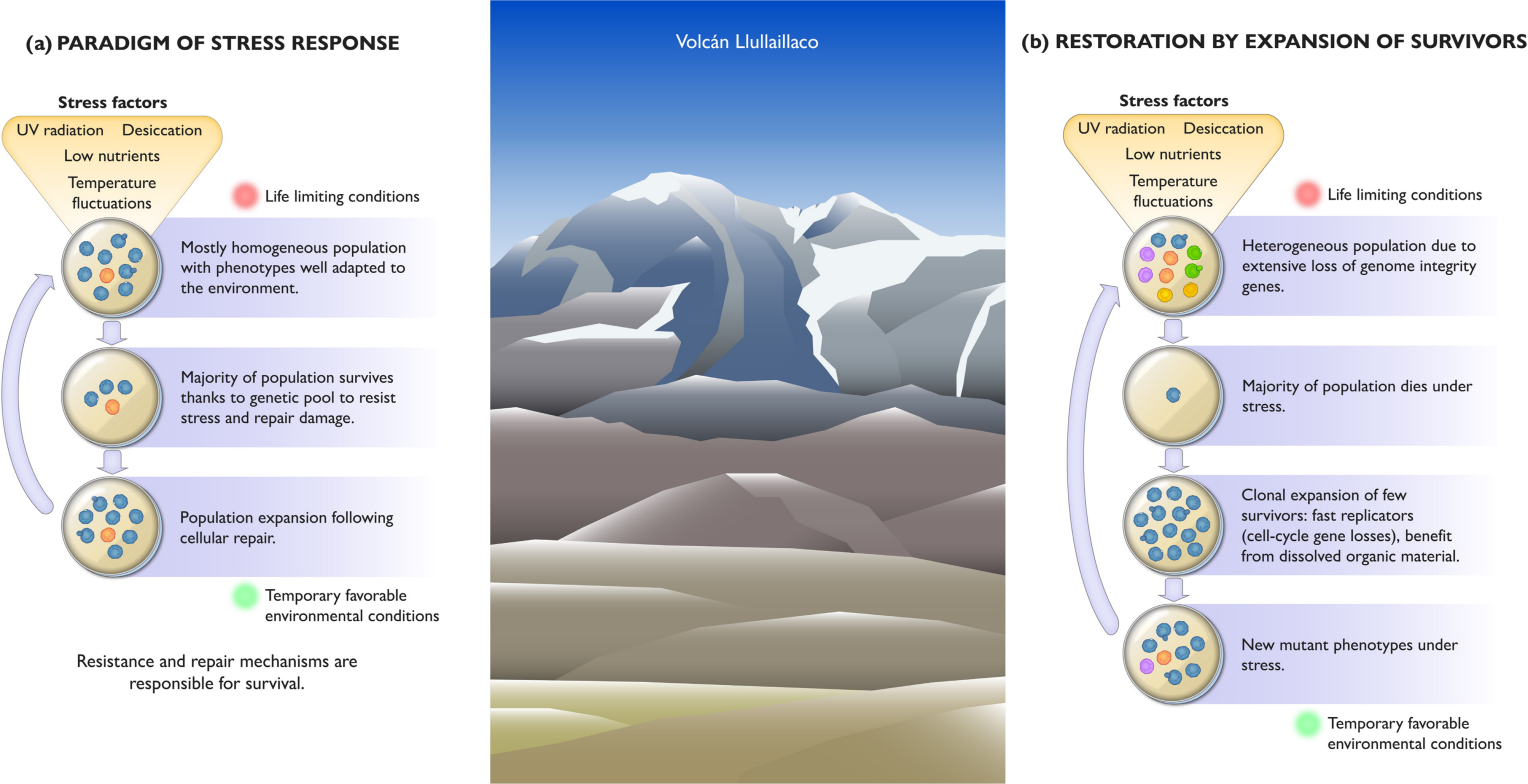
Conceptual diagram: Survival strategies in response to stress and environmental changes can be categorized into two main approaches: (a) Stress tolerance/resistance: In this strategy, the population is assumed to be mostly homogeneous, with individuals possessing phenotypes well-adapted to the environment. When exposed to stress, the majority of the population survives due to a genetic pool enriched with stress-resistant genes and repair mechanisms, including the potential for dormancy. The population expands during temporary favorable conditions after undergoing cellular repair. (b) Restoration/resuscitation: In this scenario, the microorganism exhibits a genetically and phenotypically heterogeneous population, often due to a significant loss of genome integrity genes. When a stressful event occurs, most of the population perishes, leaving only a few survivors. These survivors undergo clonal expansion and are fast replicators, benefiting from a loose regulation of cell cycle processes and lack of repair. They take advantage of organic material from the dead population to thrive. Different colors in the diagram represent distinct cell phenotypes.

The traditional view of microbial responses to stress emphasizes the importance of resistance and repair mechanisms in ensuring their survival (Fig. 8a). However, the analysis of the *N. friedmannii* genome suggests that this yeast and closely related members may be employing a different strategy. The absence of several genes related to repair mechanisms and cell cycle control may facilitate rapid population restoration by allowing a few survivors to quickly expand following stressful environmental events that kill most of the population (Fig. 8b). Restoration by expansion of survivors has been reported before in other fungi such as *U. maydis*, where recovery of population density is followed by massive environmental disturbances in part due to stress-induced cellular auto-decomposition and the recycling of the released nutrients (Kojic and Milisavljevic 2020). The ability to quickly adapt to a changing environment provides a selective advantage to microorganisms. For microorganisms in extreme ecosystems, large changes in their environments occur when they are exposed to daily extreme thermal fluctuations, stochastic nutrient availability and fluctuations in moisture and radiation levels. While the model of defective DNA repair as a universal adaptation across the *Naganishia* clade is likely an oversimplification, genomic instability is likely context-dependent. In certain environments, such as sheltered habitats with reduced stressors, or in stable ecological niches with consistent selective pressures, genome stabilization could be advantageous. DNA repair pathways may also be tailored to specific habitats—in fluctuating extreme environments, hypermutation could be tolerated or even beneficial, aiding rapid adaptation, whereas in stable environments, DNA repair mechanisms may remain conserved to maintain genome integrity. The loss of genes traditionally known to cope with DNA damage and cell cycle control is compatible with long-term evolutionary survival, and we suggest that it may be advantageous in environments with extreme abiotic fluctuations where mutator phenotypes can change rapidly due to the accumulation of DNA mutations at high frequency (Fig. 8b). Alternatively, it is also possible that *N. friedmannii* and other related species compensate for the lack of genes traditionally known to be involved in DNA damage repair and cell cycle control with unknown mechanisms, as a very high percentage (22.6%) of its genes are of unknown function.

Interestingly, sexual reproduction has not been observed in *N. friedmannii* or any of its closest relatives from Antarctica or the Himalayas (e.g. *N. antarcticus, N. bhutanensis, N. vishniacii*) (Schmidt et al. 2017). The presence of homologs in most core meiotic genes suggests that *N. friedmannii* may be equipped to perform meiosis and sexual reproduction. Nonetheless, Rec8, which is absent from *N. friedmannii*, has been shown to be required for organizing meiotic chromosome architecture in other model organisms, including the plant *Arabidopsis thaliana* (Lambing et al. 2020) and the fission yeast *Schizosaccharomyces pombe* (Sakuno et al. 2022). It remains unclear if and under which environmental conditions *N. friedmannii* and its closest relatives undergo sexual reproduction.

## Conclusion

The *N. friedmannii* genome has unveiled multiple genes associated with coping with environmental stressors, as well as an expansion of genes related to carbohydrate metabolism and transport compared to other *Naganishia* species. This is consistent with its ability to survive in landscapes that are dependent on wind-blown input of organic matter, such as the extreme high elevations of the Atacama Desert. Among the unexpected findings of this investigation was the loss of several genes involved in DNA repair mechanisms and cell cycle control, which may contribute to the extremophilic lifestyle of *N. friedmannii*. We propose that the extensive loss of repair genes might be an adaptive strategy, enabling microevolution that selects a small proportion of the population with novel traits facilitating survival in a hostile and fluctuating environment. Limited control over the cell cycle and repair may help this yeast compete in niches where rapid reproduction is advantageous, such as the extreme environments of the high Atacama volcanoes, where periods of resource availability are rare. A broader investigation should attempt to establish whether a similar pattern is found within other extremophilic fungi. Further research should aim to determine whether the loss of repair and cell cycle control genes leads to an increased mutation rate and how this impacts the yeast's ability to rapidly adapt to hostile environments by analyzing changes in allelic frequencies within a population over a short period. Transcriptomic work will also enable us to better understand responses to thermal fluctuations, a distinctive feature of *N. friedmannii's* home environment.

## Supplementary Material

foaf028_Supplemental_Files

## Data Availability

The genome assembly is publicly available via the Joint Genome Institute's fungal portal MycoCosm (mycocosm.jgi.doe.gov/Nagfr2).
