# Supplementary figures and images for: The genome of the polyextremophilic yeast, Naganishia friedmannii, reveals adaptations involved in stress response pathways, carbohydrate metabolism expansion, and a limited DNA repair repertoire
Source: FEMS Yeast Res. 2025 Jun 5;25:foaf028. doi: 10.1093/femsyr/foaf028 (PMC12204325; doi:10.1093/femsyr/foaf028)

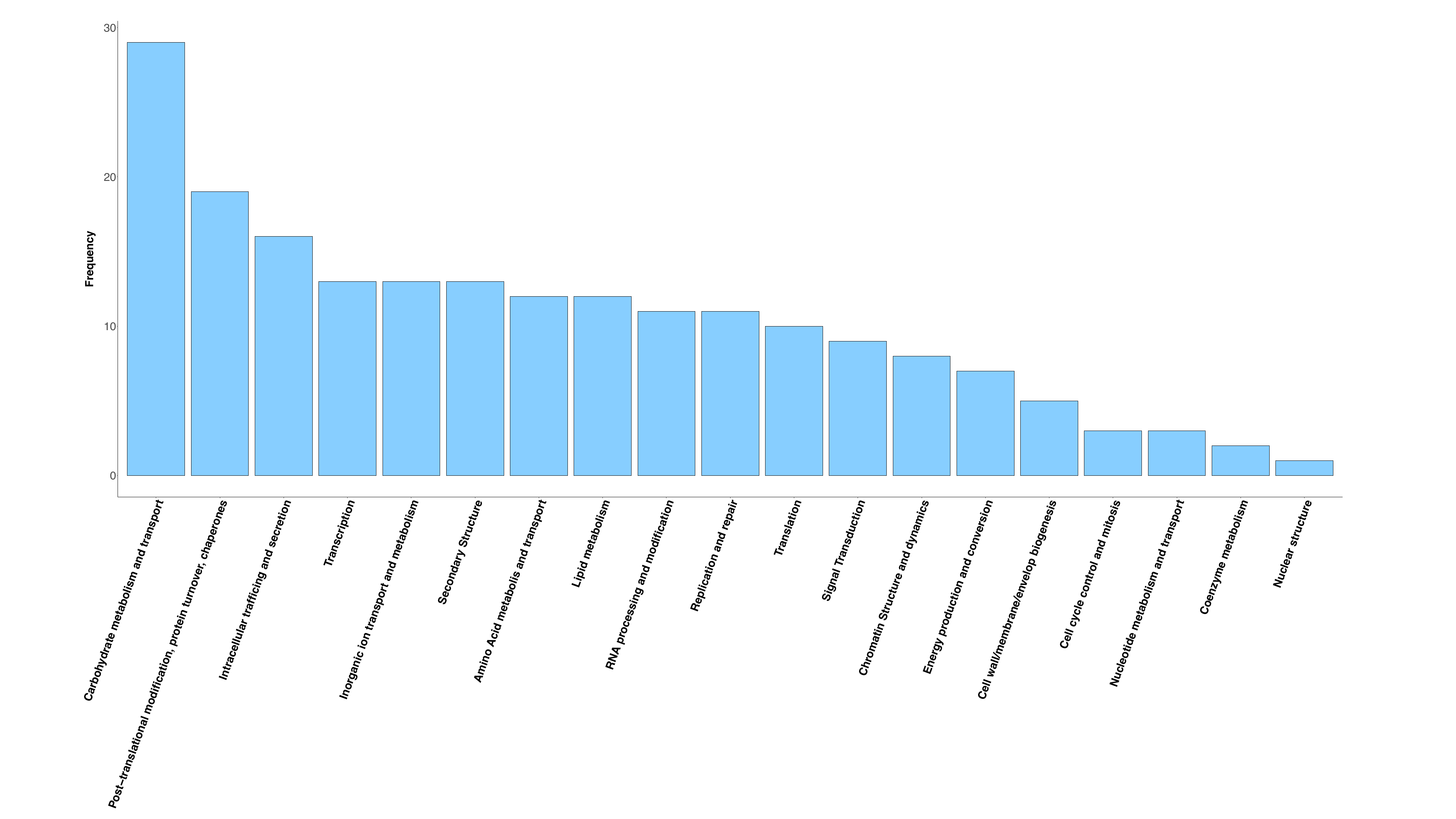

Supplement: foaf028_Supplemental_Files [file foaf028_supplemental_files.zip › Fig.S1.tif]
